# Editorial

**Published:** 1915-12

**Authors:** 


### EDITORIAL

The Atlanta Journal-Record of Medicine has its Business Office at 23 West Alabama Street where all business communications should be sent.

Remittances are payable to The Journal-Record of Medicine.

Concerning Original Communications and Editorial matters, address Richard R. Daly, M. D., 708 Empire Life Building, Atlanta, Ga.

Reprints of Original Articles will be furnished at cost price. Requests for them had best be made on the Manuscript.

Upon request, we will present, postpaid, to each contributor of an original article, twenty copies of The Journal-Record of Medicine containing such article.

### PELLAGRA.

Drs. Goldberger and Wheeler of the Public Health Service state that the cause of this disease has been found and that the way of eradication is open. Their experiments at the farm of the Mississippi State Penitentiary would seem to prove the truth of their assertion. Eleven convicts volunteered to submit themselves to the regimen indicated and for many months they received a protein free diet. In other respects they had the same life as the other convicts except that they were somewhat better housed. At the end of five months, six of them gave the characteristic skin symptoms of pellagra in addition to gastric and nervous indications that had appeared earlier. These were checked by experts of undoubted ability.

Prior to this there had been no pellagra in the institution. The only change was the deprivation of proteids and a corresponding increase in the carbohydrates in the diet. Six men had pellagra.

The editorial comment of the country is almost without exception that the disease has been discovered and thus is under our control.

It would be gratifying to join unreservedly in the chorus and to feel that our deep concern as to this disease whose ravages have been extensive and fatal to a very alarming extent, should be set at rest, but there remain some doubts to settle. We feel sure that the experimenters and investigators, themselves, are by no means ready to let others go on with the work in complete confidence as to its value.

What about the other five men? Why do they not have the disease? Why has there been this great increase in the disease in the last eight or ten years? Must one believe that only during that time and chiefly in southern countries, there have been limited diets? Of course one knows that in the northern countries, the farmers and the poorer people prepare for the long winters by salting down meats and that in other ways their diet is different from the south, but has the change been marked in this special period and increasingly so?

Does this abstention from vegetable and animal proteids obtain in the European countries where pellagra seems to originate and where it has long prevailed?

Are the investigators sure that there was no other element common to the diet of the prisoners which under the circumstances of a changed balance might have cause the irritation?

These questions are asked not to throw doubt upon the skill and good faith of the men who made the study. These gentlemen are too well known for that. But there have been other causes suggested and some of them so nearly proved that their potentiality should not be forgotten. The work done in the two Orphanages near Atlanta comprised quite as many cases and with as striking results as that in the Penitentiary. It has been published in this Journal and has always been open to investigation.

We cannot accept with alacrity the idea that a change in the balance of foods to whose character we have long been accustomed will provide the etiologic factor of which we are in search; it seems too easy. The solution of great problems and difficulties often hangs upon the simplest of matters and blindness to these little things marks off the common man from the genius that sees them, but such is not always the case.

We urge upon the students that they try similar experiments upon men under close observation and control and determine the effect of the semi-drying oils in and out of the diet. It could do no more harm than the carbohydrates and if it caused the symptoms to appear it would clarify instead of cloud the situation. There is no doubt but that symptoms do disappear when this element has been removed from the diet of pellagrins.

---

### POVERTY AND HEALTH.

Surgeon General Gorgas in his remarks upon the Factor of Poverty in Sanitation, says what everyone knows as to the dire influence of poverty, and he goes further and suggests a remedy in the increased use of the land as the best way of adding to mens' usefulness and self-support. His comments are always worthy of consideration.

Back to the farm and to the land has been the cry of the last decade and it is a good one. It is not an easy road to travel nor one to be entered into unadvisedly. Indeed, it is a matter of great public concern if for no other reason than that there is no longer land to go back to when the spirit moves the unfortunate man seeking means for a livelihood.

There is acreage enough everyone knows, but it is taken up and held unviable by owners who put posted signs at its corners. A large percentage of tillable and productive soil within practical market reach of communities is left barren because the owner does not choose to make it productive. It is held useless when needed and tax laws and vested rights permit this holding. It is held without expense because the taxing methods are gentle as applied to unproductive land.

General Gorgas suggests the remedy of taxing all tillable land so that an owner will either use it and thus give occupation to idle men or he will dispose of it to other men that will use it.

About twenty-five years ago, Henry George advocated this very thing except that he would have the title to the land resting with the State and the leasehold in the hands of the man who could make it profitable.

The problems of the unemployed are peculiarly medical ones many times and it is fitting that the leader in our sanitation should call us all to consider them and the way out. His solution indicates changes of great magnitude in our customs, but we must not falter on that account. Tremendous changes are going on in the world and none of us can avoid our part in them. There is no alibi.

